# Purtscher's Retinopathy After Cardiopulmonary Resuscitation: A Literature Review

**DOI:** 10.7759/cureus.38033

**Published:** 2023-04-23

**Authors:** Dharshini Balasubaramaniam, Pooi Wah Lott, Tajunisah Iqbal, Sujaya Singh

**Affiliations:** 1 Department of Ophthalmology, Faculty of Medicine, University of Malaya Eye Research Centre, Universiti Malaya, Kuala Lumpur, MYS

**Keywords:** cardiac resuscitation, cotton wool spot, chest compression, retinal hemorrhages, traumatic retinal angiopathy, purtscher's retinopathy

## Abstract

Purtscher's retinopathy is a rare angiopathy reported in patients with a history of severe trauma and other systemic diseases. The diagnosis is made on clinical grounds, and the severity varies. A 41-year-old gentleman with underlying poorly controlled diabetes mellitus and dyslipidemia was referred to the ophthalmology department for diabetic retinopathy screening. He denied visual complaints. Ocular examination revealed a negative relative afferent pupillary defect with a visual acuity of 6/6 bilaterally. The anterior segment examination was unremarkable. Both eye (oculus uterque, OU) fundus revealed a pink disc with a cup-to-disc ratio of 0.4 and peripapillary flame-shaped hemorrhages. Right eye (oculus dexter, OD) also showed multiple cotton wool spots along the superotemporal arcade involving zones 1 and 2 of the retina, while left eye (oculus sinister, OS) showed a single cotton wool spot along the superotemporal arcade at zone 1 of the retina. Otherwise, there were no visible retinal emboli, dot hemorrhages, or hard exudates, and the macula was normal. The retinal features were not characteristic of diabetic retinopathy. It mimicked hypertensive retinopathy, but the patient was normotensive. The absence of inner retinal thickening and hyperreflectivity on optical coherence tomography of the macula ruled out retinal vein occlusion. This prompted us to elicit further history, and the patient disclosed a recent admission for myocardial infarction in which he received cardiopulmonary resuscitation with chest compressions for seven minutes. Hence, the diagnosis of OU Purtscher's retinopathy was made, and the patient was monitored closely in the clinic. Purtscher’s retinopathy remains a diagnostic dilemma and should not be neglected in complex clinical contexts.

## Introduction

Purtscher's retinopathy is a rare angiopathy reported in patients with a history of severe trauma and other systemic diseases [1,2]. The diagnosis is made on clinical grounds, and the severity varies. Most patients are able to regain their vision without any treatment. We describe here a case of Purtscher's retinopathy in a patient following cardiopulmonary resuscitation with chest compressions and review the literature on this uncommon but interesting condition.

This article was previously presented as an electronic poster at the 38th Asia-Pacific Academy of Ophthalmology Congress, which was held in Kuala Lumpur, Malaysia, from February 23rd to 26th, 2023.

## Case presentation

**Figure 1:**
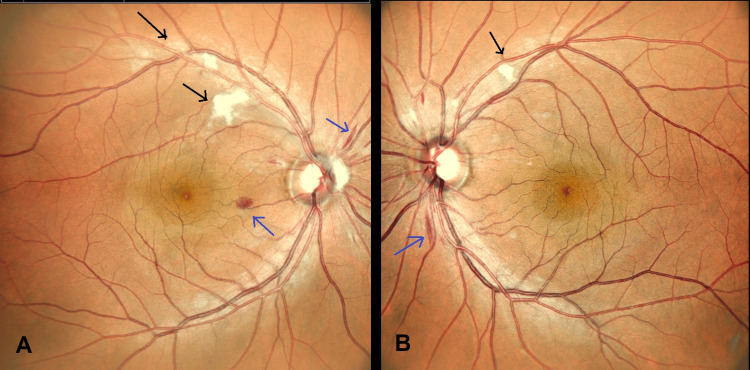
OU photo on presentation. A: OD fundus showing a pink disc with a cup-to-disc ratio of 0.4 with peripapillary flame-shaped hemorrhages (blue arrow) and multiple cotton wool spots (black arrow) along the superotemporal arcade involving zone 1 and 2 of the retina. B: OS fundus showing a pink disc with a cup-to-disc ratio of 0.4 with peripapillary flame-shaped hemorrhages (blue arrow) and a single cotton wool spot (black arrow) along the superotemporal arcade at zone 1 of the retina. OU: oculus uterque, OD: oculus dexter, OS: oculus sinister.

A 41-year-old gentleman with underlying poorly controlled diabetes mellitus, dyslipidemia, bronchial asthma, and chronic hepatitis B was referred to the ophthalmology department for diabetic retinopathy screening. He denied visual complaints. Ocular examination revealed a negative relative afferent pupillary defect with a visual acuity of 6/6 bilaterally. The anterior segment examination was unremarkable. Oculus dexter (OD) fundus examination revealed a pink disc with a cup-to-disc ratio of 0.4, peripapillary flame-shaped hemorrhages, and multiple cotton wool spots along the superotemporal arcade involving zone 1 and 2 of the retina (Figure 1A). Oculus sinister (OS) revealed a pink disc with a cup-to-disc ratio of 0.4 with peripapillary flame-shaped hemorrhages and a single cotton wool spot along the superotemporal arcade at zone 1 of the retina (Figure 1B). Otherwise, there were no visible retinal emboli, dot hemorrhages, hard exudates, or Purtscher flecken. The vessels and macula were normal.

**Figure 2:**
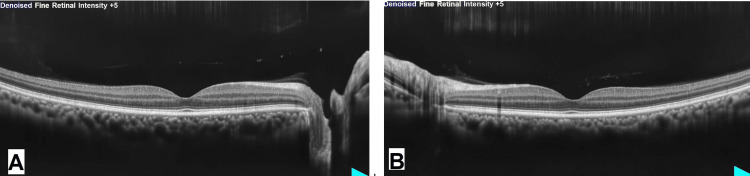
OU optical coherence tomography (OCT) of the macula on presentation. A: OD OCT macula showing a normal foveal contour with the absence of intraretinal fluid or hyperreflective foci. B: OS OCT macula showing a normal foveal contour with the absence of intraretinal fluid or hyperreflective foci. OU: oculus uterque, OD: oculus dexter, OS: oculus sinister.

The retinal features were not typical of diabetic retinopathy. It mimicked hypertensive retinopathy, but the patient was normotensive and denied being diagnosed with hypertension. The absence of inner retinal thickening and hyperreflectivity on optical coherence tomography macula ruled out retinal vein occlusion (Figures 2A, 2B).

**Figure 3:**
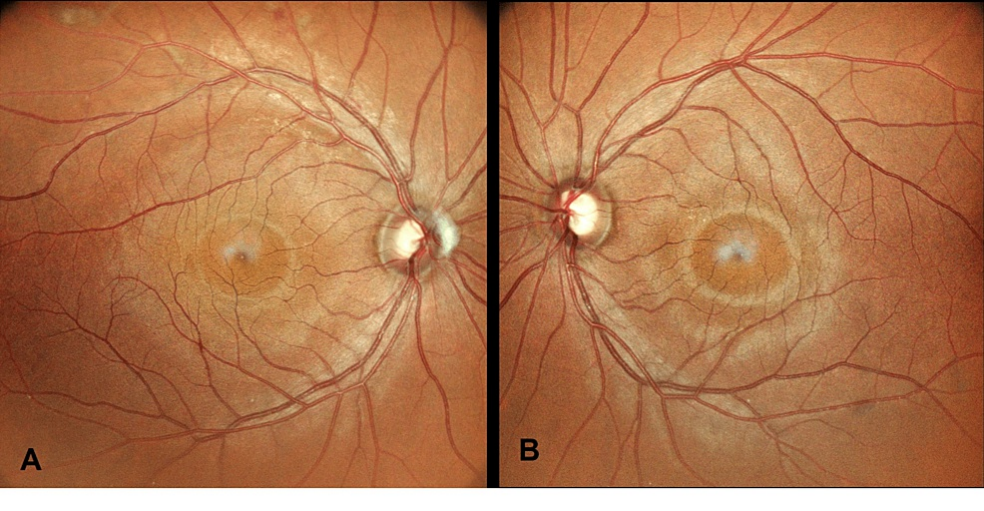
OU fundus photo six months post-presentation. A: OD fundus showing a pink disc with a cup-to-disc ratio of 0.4 and minimal residual cotton wool spots along the superotemporal arcade involving zone 1 of the retina. B: OS fundus showing a pink disc and a cup-to-disc ratio of 0.4 with resolved hemorrhage and cotton wool spots. OU: oculus uterque, OD: oculus dexter, OS: oculus sinister.

There were also no signs of infection such as retinitis, vitritis, or vasculitis, excluding an infective pathology. This prompted us to elicit further history to get to the diagnosis of the mysterious retinal findings. Patient then disclosed a recent admission to the medical ward two weeks ago for a non-ST-elevation myocardial infarction complicated with ventricular fibrillation, in which he received cardiopulmonary resuscitation with chest compressions for seven minutes. Hence, the diagnosis of OU Purtscher's retinopathy was made, and the patient was monitored closely in the clinic. Six months after presentation, there were minimal residual cotton wool spots over the superotemporal arcade of the OD, while the cotton wool spots and hemorrhages over the OS resolved spontaneously (Figures 3A, 3B).

## Discussion

Purtscher's retinopathy, also known as traumatic retinal angiopathy, is an occlusive microvasculopathy characterized by multiple retinal white areas around the optic nerve head and fovea, which may be associated with intraretinal hemorrhages [1]. It is commonly caused by chest compressions, severe head trauma, long bone or crush injuries, battered baby syndrome, acute pancreatitis, chronic renal failure, and connective tissue disorders [1-3]. Purtscher’s retinopathy is a clinical diagnosis with a presentation that usually includes unilateral or bilateral vision loss of variable severity and visual field defects [2,4]. It may be spontaneous or may occur within 24 to 48 hours of the causal pathology [2,4].

Typical fundoscopic signs include cotton-wool spots and intraretinal hemorrhages, described in 83-92% of a case series [5], which were also a presenting feature in our patient. Purtscher's flecken is considered pathognomonic but only occurs in 50% of cases [5]. Purtscher's flecken is described as multiple, discrete areas of retinal whitening in the superficial aspect of the inner retina between the arterioles and venules [2,4]. All these lesions are usually restricted to the posterior pole around the optic disc, rarely progressing beyond the midperipheral retina. Occasionally, optic disc swelling may be noted. If a visual field is performed, it generally shows central or paracentral scotomas; however, a rare presentation with homonymous hemianopia has also been documented [4].

A systematic review by Miguel et al. [6] states that the diagnosis of Purtscher's retinopathy is established in the presence of at least three of the five following criteria: Purtscher's flecken, retinal hemorrhages in a low to moderate number (1-10), cotton-wool spots, a probable or plausible explanatory etiology, and complementary investigation compatible with the diagnosis. Despite the good vision and minimal clinical findings, our patient fulfills these criteria as he presented with retinal hemorrhage, cotton-wool spots, and a history of chest compression, which is a common cause of Purtscher’s retinopathy.

Isolated case reports suggest treating Purtscher’s retinopathy with high-dose intravenous or oral steroids. Wang et al. [7] and Atabay et al. [8] reported an improvement in vision after treatment with intravenous methylprednisolone. They attributed the visual recovery to the ability of megadose steroids to stabilize damaged neuronal membranes and microvascular channels, allowing some healing of the nerve fibers that had not yet sustained permanent damage. This, however, is still debatable, as recent case reports such as Miguel et al. [6] reported no statistically significant difference in visual acuity improvement between patients receiving corticosteroids versus no corticosteroids. Gil et al. [9] also reported a favorable response to conservative treatment. A conservative approach was adopted in our case too, as the presenting visual acuity was good. Hence, he was closely monitored in our eye clinic.

A literature search was done on recent case reports on Purtscher’s retinopathy cases from 1993 to 2021. Patient characteristics, typical findings, and visual outcomes were tabulated (Table 1).

**Table 1:** Patient characteristics and visual outcome in patients with Purtscher’s retinopathy from the year 1993 to 2021. MVA: motor vehicle accident; HE: hard exudate; HM: hand movement; OD: optic disc; PF: Purtscher flecken; DBH: dot blot hemorrhages, CF: counting finger, IV: intravenous, CWS: cotton wool spot, HELLP: hemolysis, elevated liver enzymes and low platelets, SLE: systemic lupus erythematosus; BE: both eye; RE: right eye; LE: left eye.

Study	Laterality	Age	Sex	Etiology	Presenting features	Presenting vision	Final vision	Treatment
Atabay et al. [8]	Unilateral	25	M	MVA	HE, retinal hemorrhages	20/800	20/50	IV Methylprednisolone
Caplen et al. [10]	Unilateral	57	F	MVA	CWS	20/200	Defaulted	Nil
Lin et al. [11]	Bilateral	50	M	Compressive chest contusion	CWS, PF	CF	RE 0.8 LE CF	Hyperbaric oxygen
Nor-Masniwati et al. [12]	Unilateral	43	M	Valsalva maneuver	CWS, DBH retinal edema	CF	6/12	Indomethacin tablets
Ustaoğlu et al. [13]	Bilateral	25	F	Atypical hemolytic uremic	CWS, retinal hemorrhages, macula edema	BE 20/60	BE 20/20	Eculizumab, hemodialysis, and plasmapheresis therapy
Stewart et al. [14]	Bilateral	25	F	HELLP	Ischemic retinal whitening	CF	CF	Nil
Yan et al. [15]	Bilateral	18	F	Dermatomyositis	CWS, PF, DBH	20/600	20/400	IV Methylprednisolone
Yuan et al. [16]	Unilateral	41	M	Moderate head and chest injuries following MVA	CWS, retinal hemorrhages	20/200	20/50	Prednisolone acetate tablets
Tabatabaei et al. [17]	Unilateral	54	M	MVA	CWS, retinal hemorrhages large subhyaloid hemorrhage	20/400	20/100	Nil
Banach and Williams [18]	Bilateral	59	M	Gemcitabine hydrochloride for non-small-cell lung cancer	CWS, retinal hemorrhages	RE 20/50 LE CF	Nil (patient passed away due to cancer)	Nil
Hernández-Almeida et al. [19]	Unilateral	26	M	Internal carotid artery implantation	CWS, retinal hemorrhages	2/250	6/6	Nil
Campo et al. [20]	Bilateral	52	F	Acute pancreatitis	CWS, retinal hemorrhages	BE HM	RE 6/9 LE 6/60	Nil

## Conclusions

Purtscher’s retinopathy remains a diagnostic dilemma and should not be neglected in complex clinical contexts. In this report, we present a case of bilateral Purtscher's retinopathy with good vision and clinical signs that resolved without any treatment. Treatment with systemic steroids may improve the visual outcome in some patients, but there is currently insufficient evidence to support a standard treatment regime as the majority of patients recover spontaneously.
